# A Fourteen-Year Surveillance Study on the Microbiological Status of Raw Milk Dairy Products from Alpine Dairies in Northeastern Italy

**DOI:** 10.3390/foods15091479

**Published:** 2026-04-23

**Authors:** Ilaria Prandi, Alessandra Pezzuto, Andrea Massaro, Simone Belluco, Cristiano Ferrero, Juliane Pinarelli Fazion, Alberto Zampiero, Martina Ricci, Ivan Poli, Silvia Zuttion, Michela Favretti, Andrea Cereser

**Affiliations:** 1 Department of Food Safety, Istituto Zooprofilattico Sperimentale delle Venezie, Viale dell’Università 10, 35020 Legnaro, Italy; iprandi@izsvenezie.it (I.P.); amassaro@izsvenezie.it (A.M.); sbelluco@izsvenezie.it (S.B.); cferrero@izsvenezie.it (C.F.); jpinarelli@izsvenezie.it (J.P.F.); azampiero@izsvenezie.it (A.Z.); mfavretti@izsvenezie.it (M.F.); acereser@izsvenezie.it (A.C.); 2 Servizio Prevenzione, Sicurezza Alimentare e Sanità Pubblica Veterinaria, Central Directorate for Health, Social Policies and Disabilities, Friuli Venezia Giulia Region, Via Pozzuolo 330, 33100 Udine, Italy; martina.ricci@regione.fvg.it (M.R.); ivan.poli@regione.fvg.it (I.P.); silvia.zuttion@regione.fvg.it (S.Z.)

**Keywords:** raw milk, raw milk dairy products, foodborne pathogens, coagulase-positive Staphylococci, *Escherichia coli*, STEC, *Listeria monocytogenes*, small local productions, microbiological monitoring

## Abstract

Raw milk dairy products, an integral part of Italian food heritage, are the primary products of small-scale farms in mountain regions where pasture is seasonal. While raw milk dairy products offer potential health benefits, their physicochemical properties make them susceptible to foodborne pathogens. Long-term surveillance of these products is essential to safeguard consumer health. Here, we present a fourteen-year microbiological surveillance of raw milk dairy products and intermediate matrices from northeastern Italy’s alpine areas, analyzing coagulase-positive Staphylococci (CPS), β-glucuronidase-positive *Escherichia coli*, *Listeria monocytogenes*, and Shiga toxin-producing *E. coli* (STEC). The most frequently detected pathogens were CPS and β-glucuronidase-positive *E. coli*, with up to 19.6% and 51.7% of samples exceeding regulatory limits, respectively. Butter, curd, and fresh cream were the most contaminated matrices. Detection rates of staphylococcal enterotoxins, *L. monocytogenes*, and STEC aligned with European detection averages (6.7%, 2.6%, and 2.1%, respectively). These findings underscore the necessity of Good Hygiene and Management Practices, together with regular microbiological monitoring to mitigate contamination risks, supporting the safety and quality of traditional raw milk dairy products in alpine regions.

## 1. Introduction

Italy is the leading European producer of Protected Designation of Origin (PDO) cheeses. In recent years (2021–2024), Italian annual production of milk, butter, and cheese products has amounted to approximately 2,500,000 t, 100,000 t, and 1,350,000 t, respectively [1]. Alongside industrial dairy plants, small-scale farms play a key role in the production of traditional dairy products, particularly those obtained from raw milk [2,3].

Raw milk cheeses represent an important component of European food heritage, accounting for a substantial proportion of PDO and Protected Geographical Indication (PGI) products [3]. These traditional dairy goods contribute to the preservation of local traditions and support rural economies. The wide variety of Italian traditional products serves as a consistent source of income for local producers, helping to prevent the desertification of remote areas while preserving traditions and local know-how that can be passed on to future generations [3,4,5,6]. Consumers, increasingly interested in local, fresh, and natural products, highly appreciate typical dairy items, especially those made with raw milk [4,7]. Raw milk products are often perceived as more flavorful than pasteurized ones [7], as the absence of heat treatment preserves the complex microbial flora of milk, which contributes to distinctive sensory characteristics [8,9]. Additionally, milk products provide high-quality proteins, minerals, and vitamins [3,6]. Several studies have also reported potential health benefits associated with the consumption of raw dairy products, including modulation of gut microbiota [10], immune system stimulation [11,12], and a reduced incidence of asthma, allergies, and hypertension [13,14,15].

Conversely, due to their high nutrient content [16], near-neutral pH, and high water activity (aw) [17], milk represents a favorable substrate for microbial growth [3,16]. Alongside beneficial microorganisms, spoilage and pathogenic bacteria may also be present, posing risks to consumer health [10,18,19]. Consumers are not truly aware of the risks related to the consumption of raw milk products [20]. Nonetheless, milk and dairy products are recognized vehicles of foodborne disease outbreaks [21,22]. In 2024, the European Food Safety Authority (EFSA) reported 31 foodborne outbreaks related to dairy products, affecting 582 people, resulting in 30 hospitalizations and one fatality [23]. The main agents of disease are *Listeria monocytogenes*, Shiga toxin-producing *Escherichia coli* (STEC), and staphylococcal enterotoxins (SE) [9,24,25]. Milk contamination may occur through direct excretion from infected udders or through contamination from the external environment [7]. During milking, pathogens may be introduced from the udder skin, milking equipment, or operators’ hands [18,26]. Additionally, dairy products can become contaminated during the manufacturing and storage processes [7,27]. Inadequate personal hygiene and insufficient cleaning of facilities further promote the risk of pathogen dissemination [28,29].

To mitigate these risks, European legislation, collectively referred to as the “hygiene package”, establishes hygiene requirements for food production. As part of this package, Regulation (EC) 852/2004 specifically addresses small facilities located in remote areas that supply food products directly to the final consumer, including alpine dairies [30]. These facilities must implement Good Hygiene Practices (GHP) and Good Management Practices (GMP) to ensure high hygienic standards and are subject to regular microbiological monitoring by local authorities [25].

Within this regulatory framework, the Friuli Venezia Giulia (FVG) Region in northeastern Italy launched a project in 2010, regulated by Regional Law n.22 of 29 December 2010, to preserve and promote “Small Local Production Units” (SLPU). These units are local facilities dedicated to producing typical products using local ingredients, including alpine dairies used seasonally for pasturing and dairy production [5]. Producers are supported through own-check manuals and a monitoring program aimed at controlling microbiological hazards associated with raw milk dairy products.

The aim of this study is to present the results of a fourteen-year microbiological monitoring program conducted in SLPUs of the FVG region, focusing on the detection of major foodborne pathogens (FBPs) in raw milk dairy products. Additionally, the study examines the presence of microorganisms across different dairy matrices to explore the variability in microbial counts within the monitored production context.

## 2. Materials and Methods

### 2.1. Sampling Facilities and Food Products

The study was conducted in the alpine areas of the provinces of Udine and Pordenone (FVG region, northeastern Italy). A total of 59 alpine dairies were included in the study from 2012 to 2025, with up to 47 facilities sampled annually depending on production activity (Table 1). The management of these facilities varied across years, as different farmers used the dairies for seasonal pasturing and dairy production (during summer). Therefore, although the same dairy could be sampled repeatedly across different years, management could vary over time depending on the farmers using the facility.

The monitored products included dairy products made from raw milk, according to the own-check manuals. Alpine dairies are small facilities located in remote areas and do not have pasteurization or thermization systems. Cheese was produced according to the traditional practices of each Alpine dairy, with ripening periods ranging from 30 to more than 60 days.

### 2.2. Sampling Plan and Sample Collection

The matrices tested included butter, cheese, curd, fresh cream, fresh ricotta, raw milk, and smoked ricotta. The number of samples collected for each product and the corresponding microbiological analyses are summarized in Table 2. Samples were collected by local health units as part of the annual regional monitoring plan of the SLPUs Project.

In each study year, all alpine dairies active during the summer production season were systematically included in the monitoring scheme and visited once. During the visit, one sample of each available matrix was randomly collected and analyzed for the target microorganisms included in the monitoring plan. Raw milk and dairy product samples collected during the same visit originated from the same alpine dairy, but were not necessarily derived from the same batch, as the monitoring scheme was not designed to establish batch-specific links among samples. The annual monitoring plan was periodically updated according to epidemiological evidence and regulatory developments.

A sample of around 200 mL of bulk milk or fresh cream was collected in sterile tubes, while individual units of butter, cheese, and ricotta (both fresh and smoked) were placed in sterile plastic bags. Until 2019, 50 g of curd was collected in plastic tubes on the day of inspection. From 2022 onwards, curd samples (50 g) were collected by the alpine dairy operator on each production day and stored under refrigeration or in brine until collection by the veterinary service. For STEC detection, curd collected over the three most recent consecutive production days was pooled and tested, while the most recently collected individual sample was also tested for the other pathogens. All samples were stored in portable refrigerators and transported to the Istituto Zooprofilattico Sperimentale delle Venezie for microbiological analysis.

### 2.3. Microbiological Analyses

Microbiological analyses were performed following standardized reference methods, as follows.

All collected samples were analyzed for the enumeration of coagulase-positive Staphylococci (CPS) according to the ISO 6888-2:2021 [31]. Results were expressed as cfu/g. When counts exceeded 10^5^ cfu/g in finished dairy products, the presence of SE was investigated using the ISO 19020:2017 [32]. SE were not assessed in fresh cream and raw milk.

The presence of *L. monocytogenes* was investigated by PCR according to AFNOR BRD 07/10–04/05 [33], using a qualitative approach. All matrices were tested except fresh cream from 2012 to 2019 (in curd between 2016 and 2019); from 2019 onwards, analyses focused on milk.

In butter, curd, fresh cream, fresh and smoked ricotta, β-glucuronidase-positive *E. coli* was quantified between 2012 and 2019 according to the ISO 16649-2:2001 standards [34].

The presence of STEC strains was investigated in all matrices from 2012 to 2019, focusing on the O157 strain, following the ISO 16654:2001 qualitative method [35]. From 2022 onwards, the analysis was performed only on curd and extended to STEC serotypes other than O157, following the ISO TS 13136:2012 method [36] and the ISS-EU-RL-VTEC—Method 04 Rev2 2021 [37]. Curd samples from the three most recent consecutive production days were analyzed as pooled samples and, in case of a positive pooled result, each individual sample was subsequently tested to identify the positive one(s). For the purposes of the present study, only the pooled result was considered. Samples were classified as positive when cultivable STEC was isolated from PCR-positive samples, as presumptive when they were PCR-positive but culture negative. Negative samples tested negative by PCR.

For quantitative analyses (CPS and β-glucuronidase-positive *E. coli*), the limit of quantification (LOQ) for the tested matrices was 10 cfu/g. The remaining targets were investigated using qualitative approaches according to the corresponding reference methods.

For the descriptive analysis of CPS, bacterial counts above 10^5^ cfu/g were considered positive in all matrices, except for raw milk, for which a threshold of 10^3^ cfu/g was applied. These limits were set by the Decreto del Presidente della Regione 14 luglio 2011, n. 0166/Pres, which set the rules for the monitoring of SLPU products in the FVG region [38]. For β-glucuronidase-positive *E. coli*, a limit of 10^4^ cfu/g was used to distinguish positive and negative samples in all matrices, except for butter and fresh cream, where a threshold of 10^2^ cfu/g was applied. These criteria were established according to the Italian Guidelines CSR n.212 of 10 November 2016 [39].

In 2020 and 2021, the sampling program and related analyses were significantly reduced due to limitations imposed by the COVID-19 pandemic.

The heterogeneity of the analyses performed and matrices tested over the study period reflects successive regulatory updates, which shaped the annual sampling plan in response to newly acquired epidemiological data.

### 2.4. Statistical Analyses

All analyses were performed using R software (v4.3.2) [40]. Due to the biological nature of the data, microorganism counts (cfu/g) were converted to log cfu/g. Samples below the LOQ were treated as left-censored observations and, for descriptive and comparative analyses, were assigned a value equal to LOQ/√2 before logarithmic transformation. Descriptive statistics of the quantitative data included the median, 1st quartile (Q1), and 3rd quartile (Q3). The 95% Confidence Interval (C.I.) of pathogen presence in the tested matrices was calculated using the binom.test() function in R.

The statistical analysis was performed with the aim of exploring pathogen distribution across matrices. Specifically, the Kruskal–Wallis test was used to compare CPS and β-glucuronidase-positive *E. coli* counts across matrices. Statistical significance was defined as *p*-value ≤ 0.05. Significant results from the Kruskal–Wallis test were followed by Dunn’s post hoc tests with Benjamini–Hochberg (BH) adjusted *p*-values (*p*-value_adj_) to identify pairwise differences between dairy matrices. Statistical significance was defined as *p*-value_adj_ ≤ 0.05. The Dunn coefficient indicates the magnitude of the difference in bacterial loads between matrices. Positive results indicate a higher bacterial load in the first factor, while negative results indicate a higher load in the second one.

Figures were created using the ggplot2 package [41].

## 3. Results

### 3.1. Pathogen Prevalence

The prevalence of positive samples for each microorganism across the tested dairy matrices is summarized in Table 3.

CPS positive samples were most frequently detected in raw milk (19.6%), curd (17.4%), and fresh cream (13.4%). When CPS exceeded 10^5^ cfu/g in butter, cheese, curd, fresh and smoked ricotta, SE were assessed. Enterotoxins were detected in 2.5% of cheese samples. Of the five cheese samples positive for CPS, only one tested positive for its enterotoxins (20%, 95% C.I.: 0.5–71.6).

*L. monocytogenes* exhibited a low detection rate, with a maximum of 2.6% of positive samples found in raw milk.

*E. coli* was evaluated both as a hygienic criterion for the dairy process and as a zoonotic pathogen. β-glucuronidase-positive *E. coli* was used to evaluate adherence to good hygienic practices along the food chain, with a maximum prevalence in fresh cream (51.7%) and butter (45.3%).

**Figure 1:**
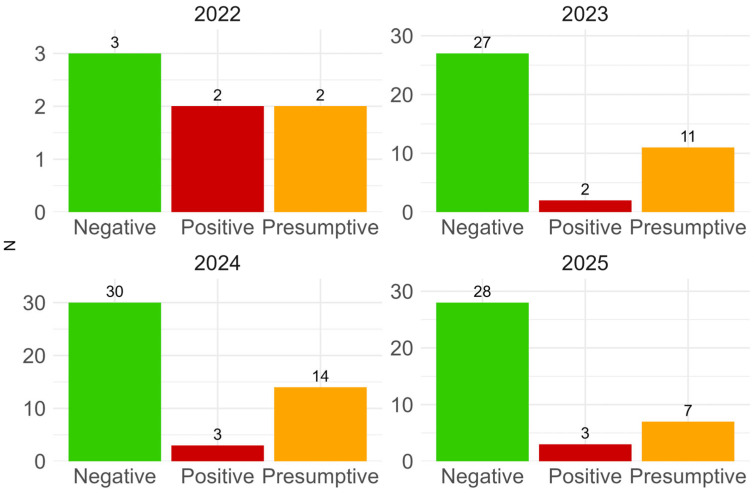
Results of STEC detection in curd samples from 2022 to 2025, categorized by the number of negative (PCR-negative), presumptive (PCR-positive but bacterial culture-negative), and positive (both PCR-positive and bacterial culture-positive on selective media) samples.

The presence of pathogenic *E. coli* O157 was assessed from 2012 to 2019, with a generally low detection rate ranging from its absence in cheese and curd to 2.1% in butter. From 2022 onwards, analyses expanded to include other STEC serotypes, focusing on curd, where a prevalence of 7.6% was detected. Additionally, 34 out of 132 tested curd samples (25.8%, 95% CI: 18.5–34.1) were identified as presumptive. The number of positive, negative, and presumptive samples for STEC strains in curd from 2022 to 2025 is shown in Figure 1.

For some microorganism–matrix combinations, only a few samples were tested and presented in Table 2 and Table 3 (e.g., SE in fresh ricotta) according to product availability in dairies during the sampling visit. These results are reported for completeness but should be interpreted with caution and were not emphasized in the discussion.

### 3.2. Quantitative Analysis of CPS

**Figure 2:**
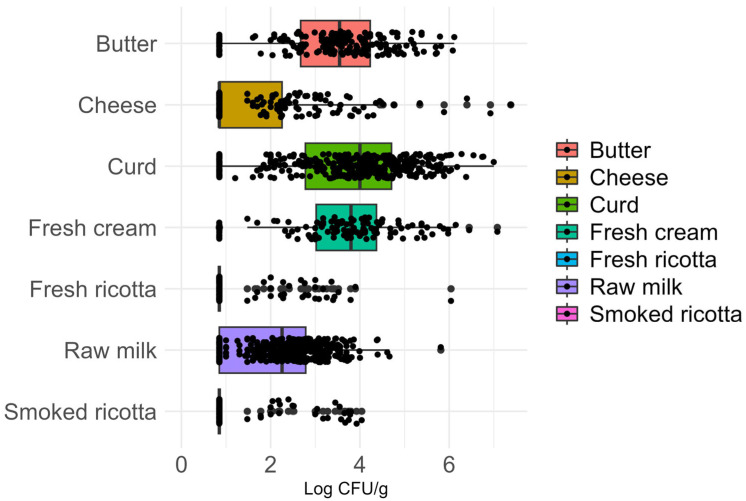
Boxplots representing the median, first, and third quartiles of quantitative coagulase-positive Staphylococci detection in the tested matrices.

Samples positive for CPS by bacterial culture ranged from a median value of 0.85 log cfu/g in cheese, fresh and smoked ricotta (IQR: [0.85–2.26], [0.85–0.85], [0.85–0.85], respectively) to 4.00 log cfu/g, IQR: [2.78–4.71] in curd and 3.80 log cfu/g, IQR: [3.02–4.37] in fresh cream. Figure 2 summarizes the median values of CPS in the different food matrices. The Kruskal–Wallis test indicated differences in CPS counts across food matrices (*p*-value < 0.001). Butter, curd, and fresh cream showed significantly higher CPS loads compared to cheese, raw milk, and both types of ricotta. Moreover, raw milk exhibited higher bacterial counts than cheese, and both had more CPS colonies than the two types of ricotta.

The statistical comparison of bacterial counts between matrices, including the Dunn coefficient and adjusted *p*-values, is presented in Table 4.

### 3.3. Quantitative Analysis of β-Glucuronidase-Positive E. coli

**Figure 3:**
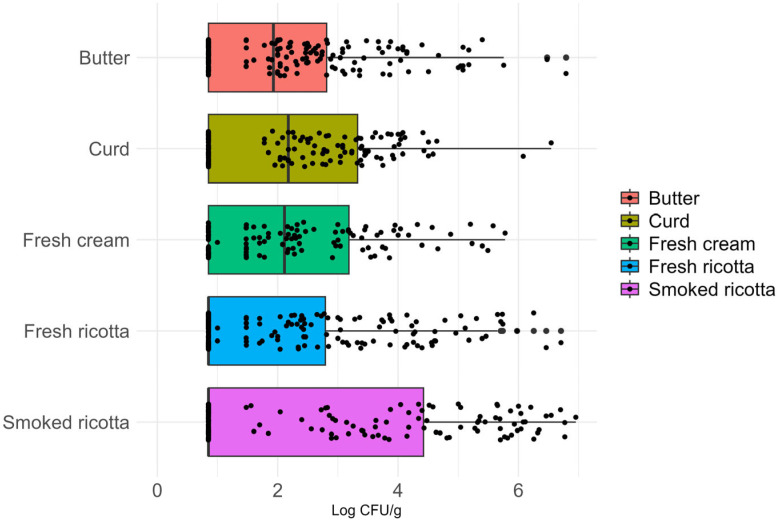
Boxplots representing the median, first, and third quartiles of β-glucuronidase-positive *E. coli* counts in the tested matrices.

Curd and fresh cream showed the highest median values of β-glucuronidase-positive *E. coli* (2.18 log cfu/g, IQR: [0.85–3.33] and 2.11 log cfu/g, IQR: [0.85–3.18], respectively), followed by butter (median: 1.93 log cfu/g, IQR: [0.85–2.81]). In contrast, both fresh and smoked ricotta showed a median value of 0.85 log cfu/g (IQR: [0.85–2.79] and [0.85–4.42], respectively). The median values of the tested products are presented in Figure 3. Statistical comparison among the tested matrices indicated differences among the matrices (Kruskal–Wallis statistics: *p*-value = 0.013). Both curd and fresh cream were associated with higher observed bacterial loads than fresh ricotta, while smoked ricotta, despite having the same median value, showed higher counts than fresh ricotta. Table 5 summarizes the statistical comparison of *E. coli* loads between the tested products.

## 4. Discussion

FBPs are microorganisms that contaminate food products and are the primary cause of human diseases associated with food consumption [16]. Animal-derived foods, particularly dairy products, are among the main vehicles of FBPs [16]. In this context, raw milk dairy products produced in small local alpine dairies could represent a potential source of FBPs [2]. However, these products also offer traditional, high-quality, locally produced goods with high nutritional value [3,6]. Preserving the continuity of these products while ensuring their microbial safety for consumers is essential. This study presents the findings of a fourteen-year surveillance study focused on primary FBPs in locally produced dairy products. It should be noted that in this study, the number and distribution of samples across the different groups of microorganisms were not constant (Table 2).

The proportion of samples with CPS concentrations exceeding the limits [38] ranged from 0.4% to 17.4% among dairy products, with 19.6% of raw milk samples exceeding the limit (the regulatory threshold for raw milk is 10^3^ cfu/g, compared to 10^5^ cfu/g for raw milk cheese). Previous studies have reported a wide range of prevalences, from 0.0% in raw milk cheeses in England [42] and Ireland [43] to 33.3% in Koryciński cheese from Poland [44] and 76.2% in Minas Frescal cheese in Brazil [45]. In the alpine region bordering Italy, Switzerland, and Austria, Berger et al. reported 16% of raw milk cheeses with CPS loads above 10^5^ cfu/g, which is higher than the 2.3% observed in our study [46]. CPS are a group of nine species belonging to the genus *Staphylococcus* spp., capable of producing coagulase and promoting plasma clotting, with *Staphylococcus aureus* being their main representative [47]. These bacteria are widespread commensals of the skin and mucous membranes of both animals and humans, but they also have pathogenic potential and are among the main causes of mastitis in dairy animals [48,49]. As a result, milk can be contaminated through colonization of the teat canal [50], and the bacteria may also be present on the udder and surrounding skin, entering milk during milking, particularly under inadequate hygienic conditions [51]. The environment and food handlers may also contribute to contamination [52]. Therefore, CPS can enter dairy products at both the primary production and processing stages, with high counts indicating poor hygienic practices [49]. In our study, the highest proportions of samples exceeding the regulatory limit were found in raw milk and curd. Likewise, the highest bacterial counts were detected in butter, fresh cream, curd, and raw milk. The sanitary quality of these products is highly dependent on the initial steps of dairy production, such as milking and refrigerated transport. Since butter and fresh cream do not undergo thermal treatments during their production, higher bacterial counts are expected. Conversely, CPS prevalence and quantity in cheese were consistently lower. This is likely linked to the reduced ability of *S. aureus* to compete with other microbial populations, particularly in the presence of active starter cultures [26,46]. Furthermore, *S. aureus* counts generally decline during ripening [9]. In addition, the cooking step in ricotta production, which reaches temperatures above 80 °C [53], is able to inactivate these bacteria (*S. aureus* grows between 7 °C and 48 °C) [54]. These processes could help explain the low detection rate in cheese and ricotta samples [55], as well as the higher load observed in other matrices such as butter, curd, and fresh cream.

The growth and spread of CPS can be mitigated at both the farm and dairy industry levels. Preventing mastitis in cows and maintaining proper hygiene of milking equipment and the surrounding environment are essential for reducing CPS loads in milk and dairy products [49]. At the processing plant level, ensuring adequate personal hygiene, controlling temperature (e.g., refrigerating milk below 7 °C), and applying multiple hurdles during production (such as rapid pH reduction, salt supplementation, proper fermentation, and the use of starter cultures) help keep CPS at low levels, preventing enterotoxin production [7,54,55].

SE are among the main causes of foodborne outbreaks worldwide [56]. These toxins are heat-stable and persist in contaminated food even after bacterial elimination [19,57]. This is particularly concerning, as only 100 ng of enterotoxins is sufficient to cause symptoms in children [58]. The toxins act on intestinal emetic receptors, triggering gastrointestinal symptoms such as vomiting, nausea, diarrhea, and abdominal cramps [16,26]. In this study, 2.5% of cheese samples tested positive for SE, consistent with the European detection rate of 2% in official cheese samples tested in 2024 [59]. Among the cheese samples with CPS counts exceeding regulatory limits (five out of 221 tested), only one tested positive for enterotoxins (20%). This percentage lies between the 6.6% described by Antoszewska et al. [44] and the 34–88.5% range reported by Zuhairi et al. in cheese samples [60].

Listeriosis is the fifth most commonly reported zoonosis in the EU and one of the most severe foodborne diseases due to its high hospitalization and mortality rates [23,26,61]. In healthy individuals, it is typically asymptomatic or causes mild gastroenteritis [62]; however, in immunocompromised patients, it can lead to severe outcomes such as sepsis, stillbirth or miscarriage, and meningitis [63]. Severe symptoms typically occur when *L. monocytogenes* reaches concentrations above 100 cfu/g, even in immunocompromised individuals [21,26], while EFSA estimates that 90% of invasive listeriosis cases occur when concentrations exceed 2000 cfu/g [64]. In the present study, we investigated the presence/absence of *L. monocytogenes* in 25 g samples by PCR. The matrix with the highest prevalence was raw milk, with 2.6% detection rate, followed by curd and cheese (1.3% and 1.4%, respectively). These prevalence rates align with previous reports at both national and international levels. *L. monocytogenes* incidence rates range from 2.2% to 3.6% in raw cow milk [65,66], and it has been detected in raw milk cheese with prevalence rates ranging from 0% to 6.5% both in Italy and Europe [67,68,69]. EFSA’s 2024 report described a prevalence of 1.6% in raw milk cheese [23].

Contamination by *L. monocytogenes* can occur at both farm and processing levels. This bacterium can proliferate in straw and improperly fermented silage consumed by dairy animals [70], and it may subsequently be shed into the environment via feces for several months or even years [26]. This environmental contamination can then be transferred to milk and milking equipment [71]. However, the primary source of *Listeria* contamination is the processing plant. Due to its ability to form biofilms, *Listeria* can resist sanitation procedures, desiccation, and nutrient deprivation, allowing it to persist for long periods in the processing environment [72,73]. *Listeria* can replicate under refrigeration and survive in high salt concentrations and low a_w_ [74], making dairy plants an ideal environment for its growth [75]. Soft and semi-soft cheeses are the most at risk due to their high moisture content [26]. In contrast, butter and ricotta generally present a lower risk of contamination during production [75]. For ricotta, a cooking step reaching 80 °C is performed [53], and the bacterium is typically concentrated in the curd rather than in whey [65]. This is consistent with our results, as *Listeria* was not detected in ricotta. However, post-processing contamination remains a concern [76], as demonstrated by an outbreak in the United States linked to contaminated *ricotta salata* [61]. Preventive measures, such as strict sanitary procedures and effective cleaning and disinfection of food-contact surfaces, are crucial to reduce *Listeria* contamination [16]. Proper GHP and GMP should be consistently applied to prevent dissemination [77], and immunocompromised individuals should be informed to avoid high-risk products to reduce infection risk [77].

*E. coli* is a commensal bacterium of the gastrointestinal tract of humans and animals and is shed into the environment via feces [18]. Its presence in food is therefore generally associated with environmental contamination by manure, soil, or contaminated water [28], often resulting from inadequate hygienic practices during production [78]. Higher incidences of *E. coli* in bulk milk have been reported on farms with poor hygienic conditions, such as failure to wash udders or maintain clean milk containers [79]. In dairy products, β-glucuronidase-positive *E. coli* is routinely tested as an indicator of the hygienic quality of the food chain. *E. coli* has a high affinity for milk fat globules, leading to a higher prevalence in fat-rich products [12]. Additionally, curd formation during cheese production acts as a physical barrier, entrapping bacteria [27]. These mechanisms could explain the high counts of β-glucuronidase-positive *E. coli* detected in butter, cream, and curd, as well as the high proportion of samples exceeding regulatory limits [39]. The bacterial concentrations observed in this study are consistent with previous reports, which described counts ranging from 4.0 × 10^2^ to 8.8 × 10^4^ cfu/g in cheese [67] and approximately 2.34 log cfu/g during the cheese-making process [27]. In contrast, whey expels without carrying *E. coli*, and the cooking process eliminates remaining strains [80]. This could explain the low bacterial loads observed in fresh ricotta compared with fresh cream and curd. The presence of *E. coli* in fresh and smoked ricotta samples exceeding limits is likely due to post-processing contamination [80].

STEC are pathogenic *E. coli* strains responsible for the third most common foodborne illness in Europe, with an increasing incidence in recent years [23]. Ruminants, particularly cattle, are the primary reservoirs and asymptomatic carriers, leading to contamination of cattle-derived food products [81,82,83]. STEC are acquired through forage, concentrates, and drinking water, and intermittently shed in feces, with excretion influenced by several factors, including animal age, diet, stress, housing conditions, herd size, and geographical area [84]. Fecal contamination of the environment can spread to teats, udders, and milking equipment, resulting in milk contamination [7,79]. Consequently, raw milk and the related dairy products represent significant vehicles for human infection [85].

The STEC group includes several serogroups, with O157 historically being the most commonly reported cause of severe human illness [85]. For this reason, it was the only serotype tested in this study from 2012 to 2019. However, non-O157 serogroups, such as O26, O45, O103, O111, O121, and O145, have shown an increasing prevalence over the years [27], responsible for about half of reported STEC outbreaks [78]. In particular, *E. coli* O26 is the most commonly detected serogroup in raw milk dairy products [86]. Although Reg. (EC) 2073/2005 does not establish microbiological criteria for STEC in food, except for sprouts [87], expert committees have recommended guidelines to reduce STEC contamination along the food production chain [88]. The General Food Law (Reg. (EC) 178/2002) supports the implementation of preventive measures against contamination and microbiological analyses on food products [89]. Within this framework, Italy issued national guidelines in 2025 addressing STEC detection, management, and mitigation in unpasteurized milk and dairy products [90]. STEC detection involves a two-step approach: PCR screening for *stx1*, *stx2*, and *eae* genes, followed by bacterial culture of PCR-positive samples [42]. However, in a significant proportion of samples, PCR positivity is not confirmed by culture [86,91], leading to results classified as “presumptive”. This discrepancy may be due to STEC concentrations below the detection limits of bacterial culture [90]. Additionally, stx genes are carried by temperate lambdoid phages, which may occur as free particles in the tested matrix or be harbored by non-*E. coli* bacterial species [42,84,86], resulting in PCR-positive but culture-negative outcomes.

In this study, only 7.6% of curd samples were culture-positive, while an additional 25.8% were classified as presumptive. Although presumptive results are considered negative in official controls, Italian national guidelines assign food business operators the responsibility to interpret these results and decide on appropriate follow-up actions when analyses are performed as self-controls [90]. Nonetheless, presumptive results should be regarded as indicative of potential contamination and warrant further investigation [42]. The *eae* gene, carried by most STEC strains, is not essential for pathogenesis [84,85], and thus, PCR-positive results are subjected to bacterial culture even in its absence. The detection rate of 7.6% STEC-positive curd samples observed in this study is consistent with European reports for raw milk cheeses, which range from 0% to 13% [84]. It is also important to note that STEC levels tend to decrease during cheese ripening [25,92] due to prolonged exposure to unfavorable pH and temperature conditions, high salt concentrations, and the antagonistic activity of cheese microbiota and lactic acid bacteria [93]. However, STEC strains exhibit higher resistance to abiotic stress factors than commensal *E. coli* strains [94] and have been detected even in raw milk cheeses with extended ripening periods [21,25]. Thus, while ripening reduces STEC concentrations, it does not guarantee the complete elimination of all STEC strains across all cheese production methods. This presents a significant public health concern, as the infectious dose of STEC can range from one to 700 viable cells [95], with as few as 50 cells capable of causing disease in healthy adults [96]. Consequently, STEC-positive raw milk products cannot be marketed and must undergo further processing or be destroyed [90]. STEC toxins are responsible for a wide spectrum of clinical manifestations, from mild watery diarrhea to severe conditions such as hemorrhagic colitis, hemolytic uremic syndrome, and thrombotic thrombocytopenic purpura [26,86]. The most severe infections primarily affect vulnerable populations, including infants, young children, pregnant women, elderly individuals, and immunocompromised patients [84]. Given the potential survival of STEC in raw milk products, these groups should avoid consumption of such products and should be adequately informed about the associated risks [90]. To reduce STEC contamination, preventive measures must be implemented throughout the food production chain, from farm-level practices to proper application of GHP, GMP, and HACCP during processing and distribution [84,90].

Changes in the monitoring plan over the years meant that not all pathogens were tested throughout the entire study period, and that not all matrices were analyzed for every target microorganism. Therefore, although the study covers a fourteen-year period, the different surveillance years should be considered only as successive phases within the same regional surveillance program. Consequently, sample size varied among matrices and pathogens, and no a priori sample size calculation or power analysis was performed, as the study reflected a surveillance-based monitoring framework rather than a hypothesis-driven experimental design. Therefore, comparisons with prevalence estimates reported in the literature should be interpreted with caution, as differences in sampling design, seasonality, tested matrices, and analytical methods may influence the observed estimates. Likewise, quantitative comparisons across matrices should be interpreted as descriptive associations within the pooled surveillance dataset, rather than as estimates derived from a fully standardized longitudinal design. In addition, for some pathogen–matrix combinations, the number of samples was very small, resulting in wide confidence intervals and limiting the interpretability of the corresponding prevalence estimates. Nonetheless, in this context, the present findings should be interpreted as representative of the monitored alpine dairy production setting within the regional surveillance framework.

Overall, this study provides a fourteen-year overview of the microbiological status of locally produced raw milk dairy products. The alpine dairies included in the study were located above 1000 m a.s.l., with some facilities situated above 1500 m a.s.l. Operating in remote high-altitude settings entails structural and logistical constraints that may limit the full implementation of GMP and GHP. In this context, European Regulation (EC) 852/2004 acknowledges such constraints and allows flexibility in the application of certain requirements for establishments in remote areas [30]. Despite these challenges, the close cooperation between the food business operators of the Alpine dairies and the food safety authority, promoted within the SLPU project, likely supported the implementation of appropriate hygienic measures during the production of traditional dairy products. Indeed, the facilities monitored within this project are subject to more extensive microbiological testing than that required by European legislation. The prevalence of samples positive for major dairy-associated pathogens was generally low and comparable to reports from other European settings, suggesting an overall satisfactory microbiological profile of SLPU products within the monitored setting.

## 5. Conclusions

This study summarizes the outcomes of a fourteen-year surveillance program on raw milk dairy products from SLPUs located in the alpine areas of the FVG region, Italy. Overall, the detection of major FBPs was generally low and broadly consistent with European data, suggesting a satisfactory sanitary profile of these traditional dairy products within the monitored production context, particularly when proper GHP and GMP are applied. These findings support the value of the SLPU project in identifying critical points and guiding preventive actions against the main microbiological hazards associated with dairy production.

Further research could benefit from integrating higher-resolution approaches, such as whole-genome sequencing, to improve the characterization of isolated strains and to better understand their distribution within the study setting. In addition, including antimicrobial resistance profiling in these traditional dairy products would further strengthen the public health relevance of the surveillance framework.

Overall, this study provides useful information on the microbiological status of traditional dairy products, and these findings may help inform future risk-based sampling strategies by identifying the matrices most frequently associated with pathogen detection.

Continued surveillance, regular operator training, and consistent application of GHP, GMP, and HACCP principles are essential to preserving both consumer safety and the cultural and economic value of these traditional dairy products.

## Figures and Tables

**Table 1:** Number of alpine dairies subjected to microbiological monitoring each year during the study period. A total of 59 facilities were monitored throughout the study.

Year	N° of Tested Alpine Dairies
2012	21
2013	24
2014	28
2015	21
2016	24
2017	23
2018	41
2019	44
2020	40
2021	42
2022	45
2023	45
2024	46
2025	47

**Table 2:** Number of samples collected and tested for each dairy product-microorganism combination during the study period.

Food Matrix	Coagulase-Positive Staphylococci (n Tested)	Staphylococcal Enterotoxins (n Tested)	*L. monocytogenes* (n Tested)	β-Glucuronidase-Positive *E. coli* (n Tested)	*E. coli* O157 (n Tested)	STEC (n Tested)
Butter	193	15	1	192	195	NT †
Cheese	221	162	157	NT	149	NT
Curd	397	70	146	144	144	132
Fresh cream	119	NT	NT	120	3	NT
Fresh ricotta	225	1	2	223	222	NT
Raw milk	449	NT	77	NT	2	NT
Smoked ricotta	178	168	175	174	175	NT
* Total *	1782	401	558	853	890	132

† NT: not tested.

**Table 3:** Prevalence of positive samples (n) for target microorganisms in the tested matrices over the study period (2012–2025). For coagulase-positive Staphylococci, a threshold of 10^5^ cfu/g was used to distinguish positive from negative samples in all matrices, except for raw milk, where a limit of 10^3^ cfu/g was applied. β-glucuronidase-positive *E. coli* was considered positive when detected in more than 10^4^ cfu/g in all matrices, except butter and fresh cream, where a limit of 10^2^ cfu/g was applied. For all other pathogens, a presence/absence criterion was used. 95% C.I.: 95% confidence interval.

Pathogens	Butter		Cheese		Curd		Fresh Cream		Fresh Ricotta		Raw Milk		Smoked Ricotta	
	n/Total	%, [95% C.I.]	n/Total	%, [95% C.I.]	n/Total	%, [95% C.I.]	n/Total	%, [95% C.I.]	n/Total	%, [95% C.I.]	n/Total	%, [95% C.I.]	n/Total	%, [95% C.I.]
Coagulase-positive Staphylococci	16/193	8.3, [4.8–13.1]	5/221	2.3, [0.7–5.2]	69/397	17.4, [13.8–21.5]	16/119	13.4, [7.9–20.9]	1/225	0.4, [0.0–2.5]	88/449	19.6, [16.0–23.6]	0/178	0.0, [0.0–2.1]
Staphylococcal enterotoxins	1/15	6.7, [0.2–31.9]	4/162	2.5, [0.7–6.2]	0/70	0.0, [0.0–5.1]	NT †	NT	0/1	0.0, [0.0–97.5]	NT	NT	0/168	0.0, [0.0–2.2]
* L. monocytogenes *	0/1	0.0, [0.0–97.5]	2/157	1.3, [0.2–4.5]	2/146	1.4, [0.2–4.9]	NT	NT	0/2	0.0, [0.0–84.2]	2/77	2.6, [0.3–9.1]	0/175	0.0, [0.0–2.1]
β-glucuronidase-positive *E. coli*	87/192	45.3, [38.1–52.6]	NT	NT	14/144	9.7, [5.4–15.8]	62/120	51.7, [42.4–60.9]	31/223	13.9, [9.6–19.1]	NT	NT	51/174	29.3, [22.7–36.7]
*E. coli* O157	4/195	2.1, [0.6–5.2]	0/149	0.0, [0.0–2.4]	0/144	0.0, [0.0–2.5]	1/3	33.3, [0.8–90.6]	1/222	0.5, [0.0–2.5]	0/2	0.0, [0.0–84.2]	2/175	1.1, [0.1–4.1]
STEC	NT	NT	NT	NT	10/132	7.6, [3.7–13.5]	NT	NT	NT	NT	NT	NT	NT	NT

† NT: not tested.

**Table 4:** Statistical comparison of the coagulase-positive Staphylococci counts in the tested food matrices. The Dunn coefficient shows the magnitude of the difference in bacterial loads between matrices. Positive results indicate a higher bacterial count in the first factor, while negative results indicate a higher load in the second. An asterisk (*) denotes statistically significant results.

Matrices Comparison	Dunn Coefficient	Adjusted *p*-Value
Butter–Cheese	11.97	0.0000 *
Butter–Curd	−1.52	0.0746
Butter–Fresh cream	−1.12	0.1446
Butter–Fresh ricotta	15.78	0.0000 *
Butter–Raw milk	8.90	0.0000 *
Butter–Smoked ricotta	14.39	0.0000 *
Cheese–Curd	−15.65	0.0000 *
Cheese–Fresh cream	−11.53	0.0000 *
Cheese–Fresh ricotta	3.89	0.0001 *
Cheese–Raw milk	−5.04	0.0000 *
Cheese–Smoked ricotta	3.14	0.0010 *
Curd–Fresh cream	−0.03	0.4890
Curd–Fresh ricotta	20.15	0.0000 *
Curd–Raw milk	13.05	0.0000 *
Curd–Smoked ricotta	18.06	0.0000 *
Fresh cream–Fresh ricotta	14.81	0.0000 *
Fresh cream–Raw milk	8.70	0.0000 *
Fresh cream–Smoked ricotta	13.74	0.0000 *
Fresh ricotta–Raw milk	−9.58	0.0000 *
Fresh ricotta–Smoked ricotta	−0.52	0.3171
Raw milk–Smoked ricotta	8.24	0.0000 *

**Table 5:** Statistical comparison of β-glucuronidase-positive *E. coli* counts between the tested food matrices. The Dunn coefficient shows the magnitude of the difference in bacterial loads between the matrices. Positive results indicate a higher bacterial load in the first factor, while negative results indicate a higher count in the second factor. An asterisk (*) denotes statistically significant results.

Matrices Comparison	Dunn Coefficient	Adjusted *p*-Value
Butter–Curd	−1.07	0.2033
Butter–Fresh cream	−1.69	0.1138
Butter–Fresh ricotta	1.47	0.1410
Butter–Smoked ricotta	−1.20	0.1914
Curd–Fresh cream	−0.64	0.3276
Curd–Fresh ricotta	2.46	0.0232 *
Curd–Smoked ricotta	−0.07	0.4724
Fresh cream–Fresh ricotta	3.02	0.0128 *
Fresh cream–Smoked ricotta	0.60	0.3056
Fresh ricotta–Smoked ricotta	−2.68	0.0186 *

## Data Availability

The original contributions presented in this study are included in the article. Further inquiries can be directed to the corresponding author.

## Abbreviations

The following abbreviations are used in this manuscript:

BH	Benjamini–Hochberg
CI	Confidence Interval
CPS	Coagulase-positive Staphylococci
EFSA	European Food Safety Authority
FBP	Foodborne Pathogen
FVG	Friuli Venezia Giulia
GHP	Good Hygiene Practices
GMP	Good Management Practices
LOQ	Limit of quantification
PDO	Protected Designation of Origin
PGI	Protected Geographical Indication
Q1	First Quartile
Q3	Third Quartile
RTE	Ready to Eat
SE	Staphylococcal Enterotoxin
SLPU	Small Local Production Units
STEC	Shiga Toxin-producing *E. coli*
